# Surgical treatment of traumatic multiple intracranial hematomas

**Published:** 2014-10

**Authors:** Chaohua Yang, Qiang Li, Cong Wu, Xin Zan, Chao You

**Affiliations:** *From the Department of Neurosurgery, West China Hospital of Sichuan University, Chengdu, China*

## Abstract

**Objective:**

To summarize our experience with the surgical treatment of traumatic multiple intracranial hematomas (TMIHs) and discuss the surgical indications.

**Methods:**

We analyzed the clinical data of 118 patients with TMIHs who were treated at the West China Hospital in Sichuan University, Chengdu, China between October 2008 and October 2011, including age, gender, cause of injury, diagnosis, treatment, and outcomes.

**Results:**

Among the 118 patients, there were 12 patients with different types of hematomas at the same site, 69 with one hematoma type in different compartments, and 37 with different types of hematomas in different compartments. In total, 106 patients had obliteration of basal cisterns, and 34 had a simultaneous midline shift ≥5 mm. Eighty-nine patients underwent single-site surgery, 19 had 2-site surgeries, and 10 patients did not undergo surgery. Based on the Glasgow Outcome Scale 6 months post-injury, 41 patients had favorable outcomes, and 77 had unfavorable outcomes. Basal cisterns obliteration was a strong indicator for surgical treatment. Single- or 2-site surgery was not related to outcome (*p*=0.234).

**Conclusion:**

Obliteration of the basal cisterns is a strong indication for surgical treatment of TMIHs. After evacuation of the major hematomas, the remaining hematomas can be treated conservatively. Most patients only require single-site surgical treatment.

Traumatic multiple intracranial hematomas (TMIHs) are traditionally divided into 3 categories: 1) different types of hematomas at the same site; 2) one hematoma type in different compartments; and, 3) different types of hematomas in different compartments.^1^ Different surgical indications have been recommended.^2^-^4^ Gruen^5^ suggested that surgical indications for TMIH should be based on the size of the lesions, their location, the presence of midline shift, and patient condition. Many factors may contribute to surgical decision-making. However, there are no well-established guidelines for the surgical treatment of TMIH. To identify the best surgical strategy for TMIHs, we performed a retrospective study of those patients with TMIH within our hospital, including analysis of CT scans, treatment modalities, and outcomes.

## Methods

We reviewed all medical records of patients with TMIH who were admitted to the West China Hospital, Chengdu, China between October 2008 and October 2011 under the supervision of the West China Hospital Ethics Board. The principles of Helsinki Declaration were followed. We utilized traditional concepts and classifications for TMIH.^1^ Hematomas in different brain lobes, but that were contiguous in the same hemisphere were regarded as one hematoma, and delayed hematomas were not included.

The inclusion criteria were: 1) age between 6 and 75 years; 2) admission to our department within 24 hours after injury; and, 3) TMIH confirmed by the initial CT scan. Exclusion criteria were: 1) history of craniotomy or any serious chronic illness; 2) penetrating injury; 3) delayed hematoma; 4) pure epidural hematoma or subdural hematoma in the ipsilateral hemisphere; or, 5) any extracranial injury that could affect outcomes. The post-traumatic mass volume measurement was based on the following equation: V=ABC/2.^6^ We evaluated the midline shifts and basal cistern status according to a previously described method.^7^ Basic treatments including sedation, analgesia, and hyperosmolar therapy were performed in accordance with the published guidelines for the management of severe head injury.^8^ An intracranial pressure (ICP) monitor (Codman MicroSensor; Johnson & Johnson Professional, Inc., Raynham, MA, USA) was used for suitable patients. Surgical indications for TMIH were as follows: 1) signs of brain herniation; 2) obvious mass effect with ≥5mm midline shift and basal cistern obliteration; 3) clinical deterioration of consciousness; and, 4) intracranial pressure (ICP) ≥25 mm Hg after mannitol treatment (1.0 g/kg). For patients with different types of hematomas at the same site, a craniotomy/craniectomy was performed at an appropriate site. All hematomas were removed via one surgical approach, and the bone flap was removed or repositioned based on surgical findings. If the brain parenchyma swelled above the inner plate of the skull, a decompressive craniectomy was performed after hematoma evacuation. In the case of one hematoma type in different compartments, or different types of hematomas in different compartments, the situation was more complicated. If one incision were sufficient to address all of the hematomas, the lesions were removed through one surgical approach; otherwise, treatment was based on the condition of the patient. If herniation occurred or was imminent, the major hematoma contributing to herniation was first removed through one surgical approach, then other hematomas were removed by another surgical approach or conservatively treated if there were no obvious mass effect, and ICP could be controlled. A large fronto-temporo-parietal craniotomy was often performed to evacuate hemispheric hematomas, a bicoronal approach was used to resect bifrontal hematomas, and a sub-occipital approach was used to remove posterior fossa hematomas. Outcome was estimated based on the Glasgow Outcome Scale (GOS) score assessed 6 months after injury; GOS 4 and 5 were considered favorable outcomes, and GOS scores <4 were considered as unfavorable outcomes.

Statistical analysis was performed using the Statistical Package for Social Sciences software, version 14.0 (SPSS Inc., Chicago, IL, USA). The Chi-square test was used to compare outcome between children and adult (Fisher’s exact test) and between difference surgical sites. Comparison of the status of basal cisterns between surgical treatment and non-surgical treatment also used Chi-square test. Statistical significance was established if *p*<0.05.

## Results

**Figure 1:**
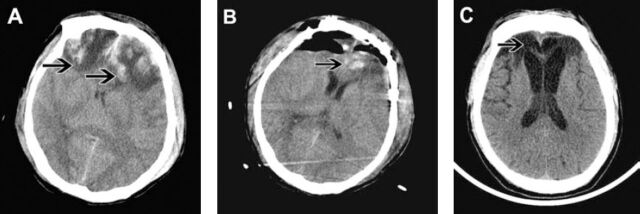
A 48-year-old male with bilateral frontal lobe contusions and hematomas that were removed through a coronal incision. Non-contrast axial head computed tomography scans showing: **A**) pre-operative scan after injury; **B**) post-operation; **C**) follow-up with good recovery.

**Figure 2:**
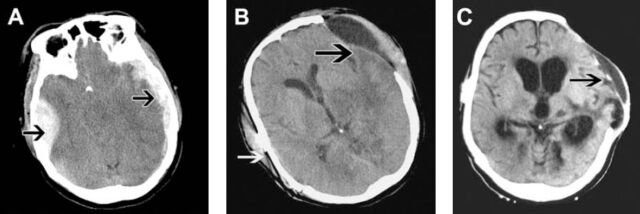
A 52-year-old male with bilateral pupil dilation underwent 2-site surgery. Non-contrast axial head CT scan shows: **A**) left fronto-temporal-parietal subdural hematoma and right temporal epidural hematoma; **B**) bilateral approach performed consecutively to remove the lesions with decompressive craniectomy performed on the left; **C**) follow-up, Glasgow Outcome Scale was 3.

**Figure 3:**
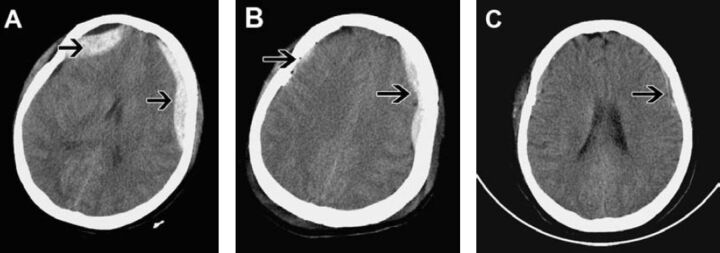
A 47-year-old female underwent single-site surgery, with the remaining hematoma treated non-surgically. Non-contrast axial head CT scans showing: **A**) pre-operative left fronto-temporal subdural hematoma and right frontal epidural hematoma; **B**) post-operative scan; **C**) follow-up, good recovery.

There were 118 patients in the study (104 males, 14 females). The patients ranged in age from 6-75 years, with a mean age of 47.1 years. Patient characteristics are summarized in **Table 1**. The TMIH classifications are listed in **Table 2**. Eighty-nine patients had hematomas ≥30 ml in volume at one site. Nineteen patients had hematomas ≥30 ml in volume at 2 separate sites. Overall, 89 patients (75.4%) underwent single-site surgery, 19 patients (16.1%) underwent 2-site surgery, and 10 patients were conservatively managed (**Tables 3 & 4**). No patients underwent 3 surgical procedures. Forty-five patients underwent ICP monitoring. The mean ICP of 25 patients was <20 mm Hg; 10 patients exhibited ICP <25 mm Hg, and 10 patients had ICP values ≥25 mm Hg. According to GOS evaluation 6 months post-injury, 41 patients had favorable outcomes, and 77 had unfavorable outcomes (**Table 1**). Among patients who were under the age of 18, only one patient experienced unfavorable outcome, compared with those patients above 18 years old. Thus, pediatric patients had better outcome (**Table 5**). Typical TMIH cases are shown in **Figures 1-3**.

**Table 1:** Demographic details of traumatic multiple intracranial hematoma patients.

Variable	n	(%)
Patients	118	
Gender (M/F)	104/14	(88.1/11.9)
***Cause of injury***		
Traffic accident	89	(75.4)
Falling	27	22.9)
Hitting	2	(1.7)
***Severity of injury***		
GCS 13-15	6	(5.1)
GCS 9-12	10	(8.5)
GCS 3-8	102	(86.4)
***Brain herniation***	39	(33.1)
Unilateral pupil enlargement	31	(26.3)
Bilateral pupil enlargement	8	(6.8)
***Basal cistern***		
Obliteration	106	(89.8)
None obliteration	12	(10.2)
***Midline shift***		
<5 mm	84	(71.2)
>5 mm	34	(28.8)
***Outcomes***		
GOS 1	33	(28.0)
GOS 2	41	(34.7)
GOS 3	3	(2.5)
GOS 4	4	(3.4)
GOS 5	37	(31.4)

GCS - Glasgow coma scale, GOS - Glasgow outcome scale

**Table 2:** Traumatic multiple intracranial hematoma patient classification based on injury type and sites.

Classification	n	(%)
***Different types of hematomas at the same site***	12	(10.2)
Intracerebral hematoma + subdural hematoma	10	(8.5)
Intracerebral hematoma + epidural hematoma	2	(1.7)
***One hematoma type in different compartments***	69	(58.5)
Intracerebral hematoma	61	(51.7)
Epidural hematoma	6	(5.1)
Subdural hematoma	2	(1.7)
***Different types of hematomas in different compartments***	37	(31.3)
Intracerebral hematoma + epidural hematoma	20	(16.9)
Intracerebral hematoma + subdural hematoma	15	(12.7)
Epidural hematoma + subdural hematoma	2	(1.7)

**Table 3:** Status of basal cisterns and surgical treatment among traumatic multiple intracranial hematoma patients.

Basal cistern status	Operation	Non-surgical
Obliteration	103	3
Not obliterated	5	7

x^2^=35.957, *p*=0.000

**Table 4:** Surgical sites and outcomes among traumatic multiple intracranial hematoma patients.

Variable	Favorable outcome	Unfavorable outcome
Single-site surgery	31	58
Two-site surgery	10	9

x^2^=1.418, *p*=0.234

**Table 5:** Outcomes between pediatric and adult groups among traumatic multiple intracranial hematoma patients.

Variable	Favorable outcome	Unfavorable outcome
Pediatric group (age under 18)	8	1
Adult group (age over 18)	33	76

Fisher’s exact test *p*=0.001

## Discussion

Caroli et al^9^ divided TMIHs into the following 3 types based on lesion predominancy: lesions with an extradural hematoma (EDH), lesions with a combination of homolateral subdural (SDH), and intracerebral hematomas (ICH), and lesions with a pure focal intracerebral hematoma (ICH). The authors reported that multiple lesions have the same prognosis as the predominant single lesion.^9^ However, Maas et al^10^ reported that patients with diffuse injuries on CT had poorer outcomes. In the current study, the percentage of severe injuries was as high as 86.4%, and the rate of unfavorable outcomes was 65.2%. In our opinion, TMIHs are more serious than single hematomas, and their outcome is worse than that reported for predominant single hematomas. Moreover, we found that pediatric patients had better outcome than adult patients. In the pediatric group, brain herniation occurred in 2 patients (22.2%, 2/9). However, in the adult group, brain herniation occurred in 37 patients (37.4%, 37/99). The patient’s condition was different, so the outcome was different.

At present, no specific indication has been proposed for the surgical treatment of TMIHs. However, we can refer to the surgical indication for single hematomas. If the ICP is not conservatively controlled, surgical intervention should be considered. In the case of TMIHs, if the major hematoma responsible for intracranial hypertension is identified and removed, the other hematomas can be treated conservatively. Surgery is indicated if the volume of a single hematoma is ≥30 ml (hemisphere lesions), this rule is also suitable for TMIHs. In our study, all of the patients undergoing surgery had hematomas with volumes ≥30 ml. Esposito et al^11^ reported that the frequency of craniotomies was 2.6% in patients with closed-head injuries. In the current study, a number of patients underwent surgery; however, if we evacuated the hematoma that was responsible for intracranial hypertension, the remaining hematomas at other sites could be treated conservatively. In fact, a large fronto-temporo-parietal or bicoronal approach can remove most of the hematomas. Some patients required 2-site surgeries via different surgical approaches, but there was no difference between one- and 2-site surgeries in terms of outcome.

A midline shift is an important surgical indicator for single hematomas and hematoma volumes correlate closely with midline shifts.^12^ In the case of TMIHs, multiple hematomas are often located in both hemispheres, and basal cistern obliteration is more frequent than a midline shift. In our study, 97.1% of the patients with basal cistern obliteration underwent surgery. Obliteration of the basal cisterns suggests a severe mass effect and intracranial hypertension. In general surgical indications for TMIHS, signs of brain herniation and clinical deterioration of consciousness indicate lower Glasgow Coma Scale (GCS). In our data, 96 out of 102 patients with GCS ≤8 underwent surgical treatment, while 12 out of 16 patients with GCS >8 underwent surgical treatment, which indicated that patients with GCS >8 may also need surgical treatment. In our opinion, the GCS is correlated with the indication of surgery, but basal cistern obliteration is a strong indication for the surgical treatment of TMIHs.

Control of intracranial hypertension is an important factor that contributes to better outcomes. Miller et al^13^ reported that the initial head CT scan findings exhibited a linear relationship with baseline ICP. When we determine the need for surgery based on the CT scan, it often means that intracranial hypertension might not be controlled with conservative treatment. Intracranial pressure monitoring can provide accurate and objective data and help us optimize ICP management. Thus, some patients can be treated conservatively or only undergo single-site surgery because of ICP monitoring. However, a second CT scan is also necessary for these patients, especially when the ICP is increased or patients have worsening mental status and pupil reactions. For those patients with brain swelling and bone flap removal, a CT scan should be performed immediately after surgery. If the total volume of the hematomas is <30 ml or the ICP is controlled (<25 mm Hg), and the patient’s condition is stable, there is no indication for surgery. At present, the benefit of ICP monitoring in severe traumatic brain injury is controversial. There were conflicting results on the role of ICP monitoring.^14^-^16^ Based on our experience, we consider ICP monitoring helpful for the management of TMIHs.

Classifying TMIHs is helpful for deciding appropriate therapies. For the patients with different types of injuries at the same site, one surgical incision can cover all of the hematomas; thus, this kind of hematoma can be considered a “single hematoma”. With respect to one hematoma type in different compartments or different types of hematoma in different compartments, the hematoma can be treated as a “single hematoma” if they can all be addressed through the same incision. If the hematomas required 2 or more surgical incisions, the surgical intervention mainly depends on the volumes of the hematomas in each possible surgical field and the ICP. After evacuating a major hematoma, the remaining hematoma should be treated conservatively if the ICP can be controlled with non-surgical treatments. In addition, we observed that 58.5% of the patients were classified as having one hematoma type in different compartments, and multiple intracerebral hematomas accounted for 51.7% of all hematomas in this study. Intracerebral hematomas accounted for the majority of our patients. Thus, the treatment of TMIHs should focus on multiple intracerebral hematomas.

In conclusion, most TMIHs are severe injuries. Surgical indication mainly depends on hematoma volume, basal cistern status, ICP, and patient consciousness level. Basal cistern obliteration is an important factor contributing to surgery, and most patients only require single-site surgery. However, the current study was a retrospective clinical analysis that included a limited number of cases. Further research should be performed to propose a widely accepted surgical indication for TMIHs.
